# Effect of a web-based chronic disease management system on asthma control and health-related quality of life: study protocol for a randomized controlled trial

**DOI:** 10.1186/1745-6215-12-260

**Published:** 2011-12-14

**Authors:** Sara Ahmed, Susan J Bartlett, Pierre Ernst, Guy Paré, Maria Kanter, Robert Perreault, Roland Grad, Laurel Taylor, Robyn Tamblyn

**Affiliations:** 1 School of Physical and Occupational Therapy, McGill University, 3654 Prom. Sir William Osler, Montreal, QC, H3G 1Y5, Canada; 2 Clinical and Health Informatics, McGill University, 1140 Pine avenue west, Montreal, QC, H3A 1A3, Canada; 3 Dept of Medicine, McGill University, 687 av des Pins Ouest, Montreal, QC, H3A 1A1, Canada; 4 Pulmonary Division, Jewish General Hospital, 3755 Côte-Sainte-Catherine Road, Montreal, QC, H3T 1E2, Canada; 5 Canada Research Chair in Information Technology in Health Care, HEC Montréal, 3000 Côte-Ste-Catherine Road, QC, H3T 2A7, Canada; 6 Education, Concordia University, 1455, de Maisonneuve Blvd W., Montreal, QC, H3G 1M8, Canada; 7 Psychiatry, Direction de la santé publique, 1301 rue Sherbrooke est, Montreal, QC, H2L 1M3, Canada; 8 Herzl Family Practice Centre, Jewish General Hospital, 3755 Cote Ste Catherine Road, Montreal, QC, H3T 1E2, Canada; 9 Canadian Patient Safety Institute, 1150 Cyrville Road, Ottawa, ON, K1J 7S9, Canada

## Abstract

**Background:**

Asthma is a prevalent and costly disease resulting in reduced quality of life for a large proportion of individuals. Effective patient self-management is critical for improving health outcomes. However, key aspects of self-management such as self-monitoring of behaviours and symptoms, coupled with regular feedback from the health care team, are rarely addressed or integrated into ongoing care. Health information technology (HIT) provides unique opportunities to facilitate this by providing a means for two way communication and exchange of information between the patient and care team, and access to their health information, presented in personalized ways that can alert them when there is a need for action. The objective of this study is to evaluate the acceptability and efficacy of using a web-based self-management system, My Asthma Portal (MAP), linked to a case-management system on asthma control, and asthma health-related quality of life.

**Methods:**

The trial is a parallel multi-centered 2-arm pilot randomized controlled trial. Participants are randomly assigned to one of two conditions: a) MAP and usual care; or b) usual care alone. Individuals will be included if they are between 18 and 70, have a confirmed asthma diagnosis, and their asthma is classified as not well controlled by their physician. Asthma control will be evaluated by calculating the amount of fast acting beta agonists recorded as dispensed in the provincial drug database, and asthma quality of life using the Mini Asthma Related Quality of Life Questionnaire. Power calculations indicated a needed total sample size of 80 subjects. Data are collected at baseline, 3, 6, and 9 months post randomization. Recruitment started in March 2010 and the inclusion of patients in the trial in June 2010.

**Discussion:**

Self-management support from the care team is critical for improving chronic disease outcomes. Given the high volume of patients and time constraints during clinical visits, primary care physicians have limited time to teach and reinforce use of proven self-management strategies. HIT has the potential to provide clinicians and a large number of patients with tools to support health behaviour change.

**Trial Registration:**

Current Controlled Trials ISRCTN34326236.

## Background

Asthma is a prevalent and costly disease with expenditures in the US alone of $648 million annually [1]. Despite the availability of effective therapies, optimal management of asthma remains problematic [2-8]. Much of the cost of asthma care is attributable to poor disease control due to non-adherence to prophylactic therapies, inadequate monitoring of disease severity, and insufficient patient education for effective self-management [1].

Effective management requires a strong collaborative partnership between patients and their care team. Asthma programs that incorporate strategies to optimise self-management reduce ED visits [9,10], hospitalisations [9,11], and healthcare costs [12,13]. However, barriers to building effective partnerships between patients and the care team include lack of patient time and engagement, reduced continuity of care, limited patient self-monitoring of symptoms, and minimal follow-up between visits by the care team [14-16].

Health information technologies (HIT) can offer novel opportunities to enhance patient self-management and patient-provider partnerships by facilitating: 1) active disease monitoring and feedback with the care team [17-19]; 2) patient education about successful adoption and maintenance of health behaviours between clinical encounters; 3) case management when problems are identified; and 3) an opportunity to share clinical information and treatment goals through patient access to their personal health record (PHR). Ongoing efforts to develop, implement, and evaluate computerized-assisted decision-support and clinical information systems for clinicians have shown promise in reducing medication errors and healthcare utilisation [20-43]. Limited work, however, has been completed in the area of decision and self-management support systems for patients [42].

### The Chronic Care Model and Patient Self-Management Support

The Chronic Care Model (CCM) [44] is an evidence-based, client-centered framework to improve care for individuals with chronic disease and provide support for their caregivers. A basic tenet of the CCM is that productive, patient-centered interactions between informed patients and knowledgeable teams across the care continuum can lead to optimal outcomes. The CCM component with the second strongest evidence base is patient self-management support, [45] which empowers individuals to develop the self-efficacy and skills needed to manage their health effectively.

#### Engaging and training patients to manage their asthma

Self-management is the individual's ability to manage the symptoms, treatment, physical, and psychosocial consequences and lifestyle changes inherent to living with a chronic health condition [46]. Beyond providing education and supportive counselling, self-management programs also teach specific skills with proven effectiveness [10,16,47,48]. The impact of self-management on health outcomes is thought to occur primarily through changes in health behaviours by providing patients with the confidence to engage in tasks and to acquire core knowledge and skills aimed at helping them better manage their health [16,49].

Optimal long-term management requires ongoing monitoring of asthma control by patients, coupled with an individualized written asthma action plan. This allows patients to quickly identify and address suboptimal disease control by adjusting medications or by contacting their physician [50-52].

Many individuals with asthma lack the necessary knowledge to effectively manage their asthma such as how to avoid common triggers and the role of medications in preventing and addressing worsening symptoms [16,53]. Often, patients do not recognize early signs of exacerbations [54,55]. It is estimated that less than 40% of patients regularly monitor their symptoms [56] and even fewer initiate their prescribed action plan at the first signs of an exacerbation [57,58]. As a result, even when an action plan is available, only a minority of patients titrate their therapy as directed [57,58]. In turn, the exacerbation may escalate to the point of requiring urgent medical attention with an increased risk of morbidity and mortality [59].

A number of studies have also found that many individuals with asthma have suboptimal levels of physical activity and aerobic endurance [60-63]. However, there has been relatively limited interest and success developing interventions to improve physical activity in asthma patients [64,65]. Reinforcement and follow-up are key predictors of long-term adherence to increased physical activity [65,66]. Support from others (e.g. health professional, peer group, trainer) can improve self-efficacy and adherence [67,68]. Proven strategies that facilitate the adoption and maintenance of increased physical activity levels include setting realistic goals, tailoring activities based on enjoyment and personal history, identifying and addressing common barriers such as time constraints, and monitoring progress [69,70].

The episodic nature of asthma symptoms and exacerbations may be a significant barrier to effective self-management. Decision research on trend perception has shown that it is difficult to recognize changes in trend in disease control when information on momentary experiences is presented in a partitioned manner [71,72]. In the context of asthma, if patients view their disease as a series of acute episodes, this impairs their ability to monitor changes in asthma control. While previous studies using simple alert systems have had little effect in improving asthma control [58], graphically representing changes in asthma control over time may increase the salience of this information. HIT tools that can improve trend perception combined with feedback from the care team have the potential to improve self-management. Web-based technology allows real time visual representation of asthma control over time [17].

Studies have also found that when patients have greater asthma knowledge of how to use their action plan effectively and are given appropriate tools to monitor symptoms and document changes, they are better able to learn and develop the skills needed to optimise asthma control [73,74]. These skills centre on problem solving when symptoms worsen, decision making to identify optimal actions (e.g., increase medicines or call the care team), and the ability to find and utilize resources [53]. Finally, given the wealth of available health information, sorting through what is relevant and appropriate is another skill that individuals need to master [16,53]. When information is easily accessible at the time it is needed (e.g., when symptoms worsen), patients are more likely to be able to act on the information. The ability to access the appropriate information, tailored to their needs and preferences, and coupled with the knowledge that they have ongoing support and feedback from the care team, can enhance confidence to develop the necessary skills to engage in essential self-management tasks.

#### Ongoing Monitoring and feedback from the care team

Another important contributor to suboptimal management of asthma is the lack of follow-up by the care team between visits [16,75]. Efforts to persist in ongoing self-monitoring and self-management are often directly related to the extent to which patients perceive that they have access to and receive feedback from the care team [75-77]. Many patients have difficulty recognizing early signs of deterioration and do not access their care team in a timely manner [78,79]. Given the high volume of patients, primary care physicians often have limited time for regular monitoring, feedback, and reinforcement. Even when face-to-face asthma education programs are available, many patients are not able to attend and often these programs do not incorporate necessary self-management skill training [80-83].

Reminders to monitor their asthma and share results with the care team have been shown to improve medication adherence [84]. Such approaches allow patients to partner with their care providers, routinely discuss trends and rhythm of their disease and share in decision-making [85]. Patients engaged in their own care may be effective catalysts for changing and optimizing clinical management of their asthma.

**Figure 1:**
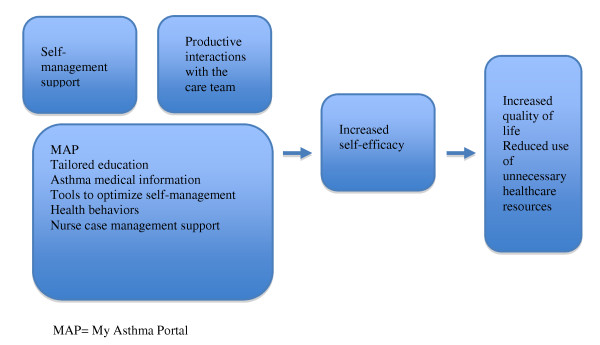
**Relationship between MAP and Health Outcomes**. MAP = My Asthma Portal.

Chronic disease case-management can effectively improve symptoms and health-related quality of life of patients with chronic obstructive pulmonary disease, diabetes, and coronary heart disease [86]. Case managers play a critical role engaging patients in treatment and providing ongoing monitoring and feedback with the clinical team [87]. To achieve this, case mangers require real time access to patients' medical records and patient reported outcomes and other information that will aid in the identification of problems as they arise. These two key principles, engaging and training patients to manage their asthma and providing patients with active monitoring and feedback from the care team, were used to develop *My Asthma Portal *(MAP) (Figure 1) and the linked nurse case-management system.

## Objectives

The objective of this study is to evaluate the acceptability and test the efficacy of using MAP linked to a case-management system on asthma control, and on asthma health-related quality of life. We hypothesize that higher rates of usage of MAP will be associated with greater improvements in asthma control and asthma-related quality of life.

## Design

The study is a parallel multi-centered 2-arm pilot randomized controlled trial (controlled-trials.com identifier ISRCTN34326236). Participants are randomly assigned to one of two conditions: a) MAP and usual care; or b) usual care alone.

### Participants

Participants are recruited from pulmonary clinics in two tertiary care hospitals in Montreal, Canada.

### Inclusion criteria

a) Males and females Age 18 to 69 years

b) Physician diagnosis of asthma and prescribed at least one rescue medication.

c) Classified as having poor asthma control by their doctor

d) Access to the internet

e) Smoking < 20 pack-years

f) Can speak and understand English or French

### Exclusion criteria

a) Diagnosis of COPD

b) Other serious medical diagnoses (e.g. lung cancer).

### Interventions

#### Control Intervention - Usual Care

Patients receive ongoing asthma care from a respirologist. An asthma nurse provides education and follow-up as needed. Topics such as the importance of avoiding triggers, taking all asthma medications as prescribed, and using the written action plan as needed. Follow-up phone calls between visits are provided by the asthma nurse, when appropriate (i.e. missed appointments, to clarify aspects of the action plan or prescribed asthma medications).

### MAP linked to a nurse-case management system

#### Initial set up and information exchange

Three systems are linked together, MAP, the nurse case-management system, and a light MOXXI electronic health record (EHR). The EHR contains drug information entered by the physician and from the Quebec provincial health database (RAMQ) including: 1) medications prescribed by the physician, and the RAMQ record for all drugs dispensed, medical services provided, ED visits and hospitalizations. Relevant data from the EHR are sent to MAP and the nurse case-management system. The MAP system also provides links to selected patient educational materials from credible online resources.

Participants are given a MAP username and password and are asked to access the internet from anywhere (home, work, library). They are asked to select the number of times they will commit to logging in per week, with a minimum log in of at least 1×/week. During the first time log in, they complete a *My Profile *page to verify basic demographic information, enter additional health information (e.g., smoking status, allergies, triggers) and select initial health goals to work on.

**Figure 2:**
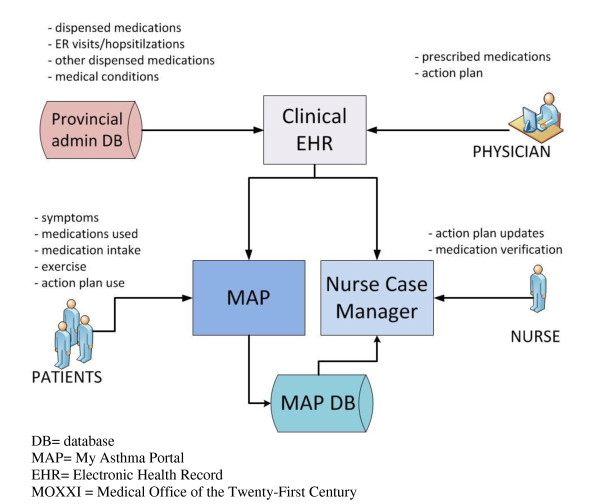
**Integration of MAP with the MOXXI EHR and nurse case-management system**. DB = database. MAP = My Asthma Portal. EHR = Electronic Health Record. MOXXI = Medical Office of the Twenty-First Century.

Once patients complete the initial log-in, their information appears in the nurse case management system. When the nurse receives a new participant file, she reviews the accuracy of the medication list, the action plan details, and clarifies discrepancies with the referring physician, if needed. As patients complete monitoring information in the MAP system (symptoms, medication adherence, action plan use, and physical activity), the information appears simultaneously in the nurse case-management system. Based on information from the EHR and participant responses to patient reported outcomes in MAP, the MAP and nurse case-management systems provide clinical decision support (described later on) to patients and the nurse. The flow of information between MAP and the other systems is presented in Figure 2.

#### Patient Portal: MAP design and features

MAP was designed to allow patients to: 1) view their personal health information (asthma medications, other health problems) 2) view general asthma health information through links to specific educational websites (learning center) and receive new asthma education tailored to participants gaps in knowledge and clinical information (e.g. educational information on the medications they are taking); and 3) facilitate monitoring and feedback to better self-manage their asthma (summary of MAP Features, Table 1). Key principals were used to guide the design of user-friendly screens and the organization and display of educational material including Instructional Design (ID) [88-92], User Centered Design (UCD) [93-95], Information Architecture (IA) [96-98], Human Computer Interaction (HCI) [93], health literacy [99-102], and usability principles [103-106]. These included organizing information in a way that would make the system intuitive for patients to use, visually appealing, and organized to facilitate key self-management activities. Colors were chosen based on their widespread application both within the medical community and within society [107] and also to help distinguish information entered by patients from that entered by the healthcare team. A user-centered iterative design process [108,109] was used to design the MAP interface. This process involved 3 nurses, 2 physicians, and 10 patients (5 for focus group, 5 for individual interviews) attending an asthma clinic (Montreal Chest Institute, asthma clinic where PE works) who were asked to provide feedback on the tool and based on the feedback changes were made to the content and layout of the system. This process continued until no more changes were suggested.

**Table 1:** Summary of MAP Features

Care Management Gap	MAP Feature
Asthma related knowledge	Tailored education by linking educational material to parts of the personal health record Learning Center

Knowledge of personal asthma medical information	Asthma Personal Health Record

Self- monitoring of asthma symptoms and health	MAP tracking system: Symptoms Medication adherence Action Plan Use and Understanding Physical Activity

Guidance for adopting optimal self-management behaviors: Adherence to preventative medication, Starting and maintaining action plan use as prescribed Initiating and maintaining physical activity Developing a partnership with the healthcare team	Create short-term behavior-change goals that are realistic, achievable and sustainable Decision Support Visual feedback of monitoring information and health behavior improvement through My Asthma Target

Ongoing communication and support from the care team	Communication and feedback from a nurse case manager

**Figure 3:**
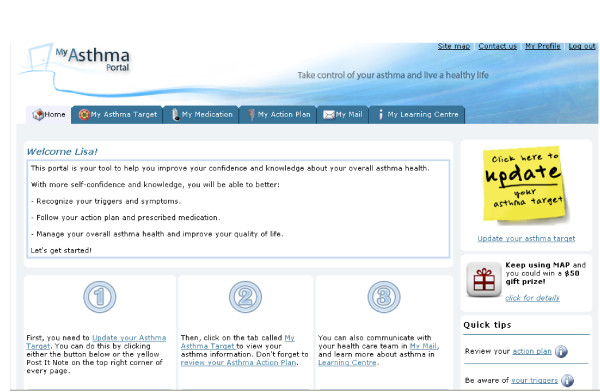
**MAP Home Page**.

The Patient Home Page highlights key elements of MAP and guides patients to areas of interest (Figure 3). The home page introduces the five core self-management areas included on the website.

**Figure 4:**
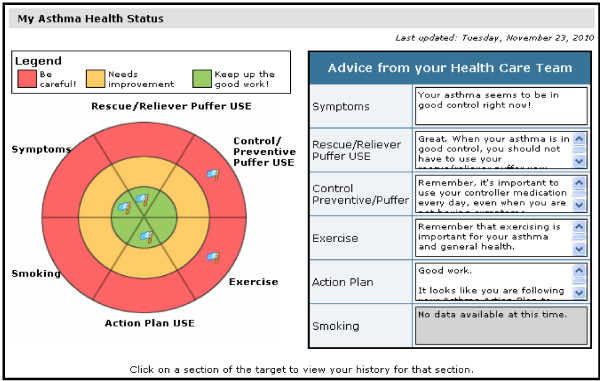
**My Asthma Health Profile**.

**Figure 5:**
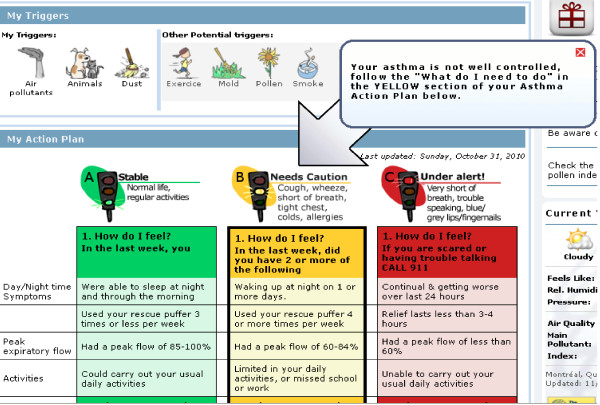
**MAP Action Plan**.

• *Update My Asthma Target*. Here, patients enter their monitoring information each time they log in. The monitoring system presents the patients' goals (previously entered in the *My Profile *page and reviewed by the nurse case manager). Each week, patients are prompted to complete the series of questions that assess symptoms, need for urgent care use (i.e., unscheduled office or ED visit), asthma medication use (medications are pre-populated in the system from the EHR), understanding of the steps of the action plan, action plan use, and physical activity. During subsequent log-ins each week, patients are only asked about medication adherence, action plan use, and physical activity information after the first login. All other monitoring information (symptoms, need for urgent care, understanding of the action plan) is assessed only once per week.

• *My Health Profile*. After each completion of the monitoring evaluation, patients are directed to the *My Health Profile to review My Asthma Target *(Figure 4) that summarizes how well they are achieving targeted goals in the areas monitored (symptom control and need for urgent care, overuse of rescue inhaler, medication adherence, appropriate use of the action plan, and physical activity) based on the updated information. This section allows patients to track asthma behaviours and receive feedback at a glance. Both color-coding (green, yellow, red) (Figure 4), and text feedback in the 'Advice From Your Healthcare Team' box are used to convey how well they are reaching targeted goals. Because examining trends over time may facilitate problem identification and promote health behavior change, graphs that show longitudinal trends over a 6 month period in symptoms, use of rescue medication, medication adherence, and number of steps walked (as an indicator of physical activity) are presented. Each graph also indicates the date on which a participant had a respiratory-related ED or urgent care visit to help link participant behaviors with relevant outcomes.

• *My Medicatio*ns. Because proper use of medications is central for optimal asthma control, a separate tab was created that lists the name of each medication, type (controller, rescue), and how it should be used (i.e., frequency and dose). A separate tab is also available where patients can view their color-coded action plan (*My Action Plan*) (Figure 5). Given the importance of the environment and avoiding stimuli that trigger exacerbations, the participant's asthma triggers are highlighted across the top of the action plan.

• *My Mail*. To facilitate communication and partnership building with the nurse case-manager, a separate tab was created to allow patients to email and receive messages from the nurse. The email view was designed to look similar to other common email applications in terms of the functions for creating and sending an email, and folders.

• *Learning Center*. Here, patients can view a table of contents of links to external sites gathered from legitimate sources of asthma and health educational information that were selected by the research and clinical team. Note: *Links to *educational materials *are also *directly integrated within the main content of the site to facilitate use. Throughout various parts of the portal, patients have information links that redirect them to specific content in the learning center thereby tailoring the educational material they are presented to their personal health information. For example, each medication has an information button that will direct the participant to the Quebec Ministry of Health Drug Database site http://www.guidesante.gouv.qc.ca which offers a description of the medication, intended use, side effects, along with potential interactions with other drugs or health conditions. Similarly, if patients indicate that they do not understand their action plan (when they respond to monitoring questions) they are taken to a web page that describes an action plan and its importance for asthma control.

#### Nurse Case Management System Design and Features

The nurse case-management system was designed to: 1) Quickly identify patients that may require immediate care; 2) Collate relevant medical and monitoring information for each participant; 3) Document participant management information, including interactions by phone and through email, and interventions and advice provided during interactions.

**Figure 6:**
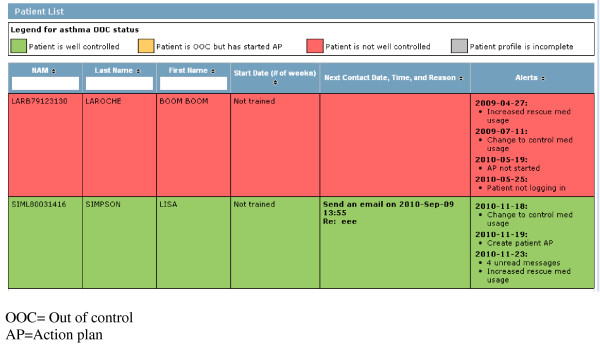
**Nurse Case-Management Triage Patient List**. OOC = Out of control. AP = Action plan.

• *Participant List*: The first view in the nurse case management portal is a participant triage list color-coded and ordered by urgency with respect to participant control status (i.e. those classified as not having good asthma control and not using their action plan will appear first) (Figure 6). The nurse's dashboard also includes a summary of alerts related to asthma monitoring (described below) and sorted by date.

• *Participant EHR*: Once the nurse clicks on a participant in the list, the nurse has access to the participant's EHR and information from MAP, including: physician name and contact information; history of symptoms and urgent visits; written action plan; asthma medications listed by the participant, and medications prescribed and dispensed; patients' monitoring information (same view as seen by patients under the *My Health Profile*: symptoms, need for urgent care (unscheduled visit or ED visit), daily use of asthma medications currently prescribed, participant comprehension of the action plan and action plan use, and physical activity); all email exchanges between the nurse and participant; clinical notes section where the nurse documents participant care and support assessments and interventions; activity log that shows all previous participant case-management activities and clinical notes on the nurse portal ordered by date.

#### Business Logic Engine for My Asthma Target and Alerts

##### Patient Alerts

The first line of feedback to patients is generated automatically from the MAP database system (Figure 2) using monitoring information entered by the participant and information from the EHR system. The business logic for the color-coding and advice presented to patients is summarized in Table 2. An alert is sent to patients via email when the system identifies that a participant's asthma is poorly controlled and the participant indicates they have not initiated use of their action plan, if the nurse has updated the action plan, or if they have not logged in for 7 days (Table 3). The participant is given an opportunity to act on these alerts and if the flag of a problem for any of these areas remains for 48 hours, the alert status is escalated to notify the nurse case-manager.

**Table 2:** Monitoring business rules

	Green	Yellow	Red
Symptoms	No symptoms	1 symptom	2 symptoms and/or emergency visit

Overuse of rescue puffer*	P < 150	250 > P ≥ 150	*P *≥ *250*

Adherence to preventative medication#	n/d ≥ 90%	90% > n/d > 70%	n/d < = 70%

Exercise†	number steps ≥ average	number steps > 2/3 of average	number steps < 2/3 of average

Action Plan Understanding%	"Yes" to all questions	N/A	"No" to at least one question

* (P = number of puffs corresponding to all FABA and combivent dispensed over last 3 months)

# (quantity dispensed/3 months) [144]

† Calculated once exercise monitoring questionnaire is completed 4 or more times (average is the mean value for the first three entries, × = the last steps entry)

%Action Plan questions: do you understand what to do in your action plan?; Are you able to describe the signs or symptoms you have when your asthma is getting worse?)

**Table 3:** Alerts to Patients and Nurse Case Manager

Event or trigger	Alert to patient	Alert to nurse
**Patient Not logging into MAP**	Emails sent at 7 days and 14 days since the last log-in to the patient.	Alert to nurse at 21 days since the last log-in.

**Asthma Out of Control (OOC) and Action Plan Use**		

Patient OOC and action plan not started:	Email to patient within 24 hours of OOC status detected	Nurse receives alert "AP not started" alert (after 48 hrs) If patient starts action plan: Patient turns yellow in case management patient list and stays yellow for 14 days.

Nurse updates action plan of a patient:	Email to patient and pop-up when patient logs-in	

Patient indicates not understanding action plan		Nurse receive alert and contacts patient

Add/change medication: Patient adds or changes medication in asthma target questionnaire		Nurse receives "create/review action plan" alert If it is the patient's first entry of medication the alert is an "Initial drug list" alert

Preventative medication monitoring		Increase or decrease of intake triggers alert to nurse

Rescue medication monitoring: FABA, oral corticosteroid		Increase triggers alert to nurse

Discrepancy in medication detected: Discrepancy between what is prescribed and what is dispensed	After 1 week an email is sent to the patient	After 2 weeks an alert is sent to the nurse

Change in medication detected: Physician enters change through MOXXI for patient's asthma medication		If, when the patient fills out the monitoring questionnaire, patient does not update medication change, an alert is sent to the nurse

##### Nurse Alerts

Upon receiving this notice, the nurse follow-ups with the participant within 24 hours through the MAP mail system or by telephone. Additional alerts are sent to the nurse when a new participant is enrolled and needs to have their action plan reviewed, the participant indicates that he/she does not understand his/her action plan, or there is a change in the way a participant is taking their medication based on the medication adherence monitoring assessment (Table 3).

### Measurements

#### Participant Demographics

Demographic information such as participant sex, age, and indicators of social economic status (e.g. household income) will be abstracted from baseline questionnaires.

### Primary Outcomes

**Asthma control **will be evaluated by examining overuse of rescue fast acting bronchodilators (beta2-agonists) (FABA) (i.e. participants will be classified as overused yes/no). Excessive use of rescue fast acting bronchodilators was included as an indicator because it is associated with an increased risk of hospitalization and death from asthma [110], and is defined as the dispensing of more than 500 doses of the most commonly prescribed fast acting beta2-agonist salbutamol 100 mcg, 2 inhalations at the time, or the equivalent for other fast acting bronchodilators in the last six months of follow-up. Doses dispensed will be based on quantities recorded in dispensed prescriptions from the RAMQ and private pharmacy prescription files. The maximum acceptable use of fast acting beta2-agonists is derived from recommendations in the guidelines that allow up to 3 doses per week and a daily dose for the prevention of exercise induced symptoms [51]. Previously developed methods will be used to adjust quantities dispensed for prescriptions filled before and immediately before the end of the item window for assessment [111].

#### Asthma Quality of Life

The Mini-Asthma Quality of Life Questionnaire (Mini-AQLQ) was developed to measure the four functional impairments that are most important for adults (symptoms, emotions, exposure to environmental stimuli and activity limitation) [112,113]. It has 15-items measured on a 7-point Likert scale. The Mini AQLQ is widely used in asthma clinical trials because it has good internal consistency (interclass correlation coefficient = 0.83), and a strong cross-sectional correlation with the 32-item AQLQ of 0.9), and moderate level correlation of 0.69 (both cross-sectional and longitudinal correlation) with the Asthma Control Questionnaire [112,114], and it is sensitive to change (reliability index = 0.97) [112].

### Secondary Outcomes

#### Acceptability and attitudes toward the web portal

The Technology Acceptance Model (TAM) [115-127] questionnaire will be used to assess acceptability and attitudes toward the web portal. The instrument was found to be reliable and valid (convergent, discriminant) for measurement of perceived ease of use and usefulness [121,123,128-130]. Reliability coefficients range from 0.92 to 0.98 for perceived usefulness and 0.88 to 0.94 for perceived ease of use [121,128]. Perceived usefulness and ease of use were significantly correlated with both self-reported current usage (r = .63, r = .45) and self-predicted future usage (r = .85, r = .59) in two different populations (users within IBM Canada's Toronto Development Laboratory, evening MBA students at Boston University) [129].

Usage rates of the system will also be assessed by examining automated audit trails (logs), which will include the frequency of use defined as the number of minutes patients spent logged into the system/week. Patterns of usage will include the number of days/week and times that patients logged in, and which features of the system they used including number of messages sent to the asthma nurse. Additional open-ended questions will be asked to receive further feedback about the usefulness of the system and how it fits with individuals' self-management practices.

**The Chronic Disease Self-Efficacy Scale **[131], has been shown to have adequate psychometric properties in patients with chronic arthritis and several conditions including hypertension, diabetes, congestive heart failure, recent myocardial infarction, major depression, and/or depressive symptoms [132] and was adapted to assess asthma self-efficacy. The test-retest and the internal consistency reliabilities were respectively 0.77 and 0.97 in an asthma patient population [133]. In the chronic arthritis population, items for test-retest reliabilities ranged from 0.71 to 0.85; all the correlations for construct and concurrent validity were significantly different from zero (p < 0.01): r = -0.35 to -0.68 for baseline self-efficacy with baseline health status, r = -0.32 to -0.68 for baseline self-efficacy with 4-month health status, r = -0.48 to -0.73 for 4-month self-efficacy with 4-month health status; r = 0.61 for concurrent validity (perceived performance and actual performance) [131]. Patients rate their level of confidence, 1 = not confident at all, 10 = very confident. Level of confidence will be rated with respect to: taking medications as prescribed; items that are behaviour-specific related to recognition and appropriate management of deteriorating asthma symptoms including ability to identify signs of deterioration and need to initiate the prescribed action plan; capacity to keep a healthy diet; ability to do exercise or physical activity on a regular basis.

#### Medication Adherence

Adherence to controller asthma medications will be evaluated by comparing medications prescribed to medications dispensed based on the prescription claims in the RAMQ system. This will be calculated using validated methods we developed to adjust quantities dispensed for prescriptions filled before and immediately before the end of the time window for assessment [111].

#### Health care Utilization

Asthma-related ED visits or hospitalizations will be abstracted from provincial database (RAMQ) using validated algorithms [134] which lists information on the date, type, provider, and location of service delivery (e.g. inpatient, emergency, clinic) for all medical services remunerated on a fee-for-service basis (approximately 86% of all services) [135].

Resource Utilization: "Information on the time spent by the nurse case manager (type of call, who is making the call, duration and reason) will be recorded, and will allow us to address health service research issues and knowledge translation to other practitioners and health care organizations. All health-care resources used during the 6-month follow-up will be considered. Intervention costs will include resources used to develop the web-based tool, train patients and nurses to use the system, administer the intervention (time spent for research data collection will not be included). Real time spent with each patient will be carefully documented by each study case manager. Case manager salary costs will be estimated based on the average pay scale of the Federation of Nurses of Quebec. Physician fees will be based on those set by the Provincial Health Insurance Board [136]. "Healthcare utilisation": Information from the provincial health insurance program administrator, RAMQ, will be obtained on asthma-related ER visits or hospitalizations. Total cost per ED visit will be based on data from earlier studies conducted in the province of Quebec [137,138]. ED visit will be a separate resource item from hospitalization. Hospitalization costs will be based on a Quebec index called the Niveau d'Intensité Relatif des Ressources Utilisées (NIRRU)" [139].

### Sample Size

The primary outcome for this study is change in asthma control. We hypothesized that use of MAP would be associated with a 10% reduction in the proportion of patients classified as being in poor control. Based on MOXXI-II results, we have estimated that 19% of asthma patients will either be seen in the ED for respiratory-related problems, or use greater than 500 doses of fast-acting beta2-agonists in a 6-month period. Thus the intervention would need to reduce the proportion of asthmatics in poor control to 9% to be considered to be sufficiently effective for policy and practice adoption.

Thus, to detect a 10% difference in proportions, [140] with 80% power using a cutoff for statistical significance of 0.05, the sample size needed is 67 participants in total. To account for attrition and loss to follow-up, we will recruit an additional 13 subjects for a total of 80 participants, and 40 participants per arm [141].

This sample size will also allow us to detect a minimal important difference of .5 on the AQLQ [113,142], using the Mini-AQLQ a difference as large as 0.97 (SD .61) was found to be clinically meaningful and significant [114,143-145].

### Recruitment

Potential participants are informed about the trial by their respirologist or the nursing staff. For individuals who agree to participate, the research coordinator further describes the study, responds to questions, and obtains informed and written consent.

### Study Procedure

#### Randomization

Treatment allocation is done by random permutation within blocks with block sizes of 4 and 6. Randomization is stratified by center. The randomization is done independently of centres, coordinator, and clinical staff. The randomization is performed with a randomization algorithm developed as part of the McGill University clinical and health informatics research tool kit. The relevant information is only accessible to an employee of McGill University Health Center who is not involved in the trial. After the inclusion of a participant in the trial and the baseline assessment, the study coordinator receives information concerning the allocation of patients to the intervention or control arm by sequentially generated logs through the study coordinator online system. Therefore, the fidelity of allocation is guaranteed.

#### Statistical methods

Descriptive statistics will be used to evaluate differences in the baseline characteristics of participating patients in the two arms of the trial. Study hypotheses will be tested using an intention-to-treat analysis, whereby all consenting patients who were randomized during the accrual period will be included in the analysis. Analysis of the primary outcome, the proportion in each group that are in control and out of control based on doses of fast-acting beta2-agonists, will be compared using a chi-square test. Changes on the Mini-Asthma Quality of Life Questionnaire (AQLQ) will be compared by independent sample t -tests, and differences between mean change scores will be expressed with 95% confidence intervals (CIs). To deal with missing data we will conduct a sensitivity analysis using multiple imputation [146,147] to explore the effects of missing data on the results. Changes in other secondary outcomes, including COPD HRQL, will be evaluated using t-tests for continuous variables and chi-square for categorical variables [141], such as individual items on the TAM that asked about perceived benefit of features of the system and intention to use the system. While our sample size will limit our ability to conduct extensive multivariate analyses, we will assess which characteristics are associated with changes in the primary outcome using multivariate regression analyses with selecting variables in each model and adjusting for potential effect modifiers, (e.g. self-efficacy, adherence, age, sex).

#### Study Timeline

Recruitment started in March 2010 and the inclusion of patients in the trial in June 2010. Follow-up evaluations are conducted at 3 months, 6 months, and 9 months after randomization.

#### Description of Risk

The risks to patients are judged to be minimal as the intent is to facilitate the implementation of evidence-based self-management where the benefits have already been shown to outweigh the risks of treatment [12,13,29,39,113,148], and system-generated advice to patients is monitored by an asthma nurse. A three phase quality assurance process is used to detect bugs whereby a study respirologist (PE) reviews the recommendations generated to ensure their appropriateness and suggests changes to the software if needed.

#### Ethics Principals

The study is being conducted in accordance with Medical Research Council Ethics Guidelines. Study participation is entirely voluntary and participants are advised they can withdraw from the study at any time without provision of reasons and without negative consequences for their current or future medical care. Ethical approval and scientific review for this study was obtained from the McGill University Health Center (IRB review number A10-E36-08B).

#### Informed Consent

Prior to study participation patients receive written and oral information about the study process and required time commitment. Potential benefits (mainly to society) and risks are explained in detail. All individuals who wish to be a participant must sign the MUHC approved consent form. In case of study discontinuation the participant will be asked whether they agree to allow all information collected up to the time of withdrawal to be used.

#### Data security/disclosure of original documents

Information collected during the study will be kept in secured offices. Information collected will only be used for the purpose of answering the research objectives of this study. Information provided to fulfill study requirements is not accessible to the nurses and physicians at the recruiting centers. Completed study questionnaires are mailed directly to the coordinating study office by the participants. As per Ethics Review Board regulations, data will be kept for 7 years after termination of the study.

All study related data and documents are stored in a protected central server of McGill University, and paper versions locked in filing cabinets in the McGill offices of the principal investigator (SA). Only investigators and staff associated with this study can access the respective files. Intermediate and final reports are stored in the McGill University office of the principal investigator.

## Discussion

Asthma represents the largest segment of respiratory diseases among Canadians with an associated cost of $306 million per year. When it comes to asthma, ongoing team care and follow-up is often limited and fragmented [149], and case- management is typically available only in specialized centers. Health information technologies provide a unique opportunity to facilitate asthma management by providing a means for two way communication and exchange of information between the patient and care team. MAP provides patients with timely access to their health information coupled with case-management, presented in personalized ways that can alert them when there is a need for action, and may empower them to self-manage more effectively and facilitate and reinforce health behaviour change.

The main focus of this trial will be on evaluating the benefit of MAP on asthma control and health-related quality of life. The increasing importance of multidisciplinary care in chronic disease gives reason for the implementation of the case manager who can assume the role of a coordinator and act as an interface between the different disciplines. One of the concerns is the increased demand on case manger time outside of the clinical encounter. We have designed the case manager system in a way to minimize needed time on the nurse's part and to facilitate access to information. Future work will examine the cost implications of providing nurse case management via information technologies through a patient portal compared to traditional care approaches.

Another potential challenge may be identifying the training needed of individuals who will use the system. While some patients are more intuitively inclined to learn how to use new technologies, some require more training time and support than others. This will be monitored through the trial to identify participant profiles that will inform future design of systems like MAP and to streamline the training process.

With any web-based tool a further concern is the differential access and comfort of participants with technologies in general for older, poorer, and less educated people who are less likely to adopt the web-based system described here [150]. The implications are that we may recruit younger and more educated individuals who are more inclined to have access to computers and thus the results may not be immediately generalizable to other patient groups. Further, MAP is a multi-faceted intervention and the results from this trial will not be able to attribute any potential improvement in outcome to a specific component of the intervention. If benefits are found from this pilot, future studies will incorporate comparison groups to identify specific components of MAP that are necessary for improving outcomes.

As new technologies are developed and their benefits rigorously evaluated, the provider-patient workflow will continue to evolve. The challenge remains to develop integrated systems of care that permit easy access to information, enhance collaboration between clinicians and patients, and emphasize a patient-centered approach to care by addressing the self-management needs that are most relevant to patients. Individual access to patient health portals coupled with self-management tools and efficient feedback from the care team represents one such new paradigm of care. Results from our study will provide clinical trial evidence for the clinical impact of a web-based chronic disease management system and a new structure for collaborative care.

## Trial status

Ongoing

## List of abbreviations

HIT: Health information technology; MAP: My Asthma Portal; ED: emergency department; PHR: personal health record; CCM: Chronic Care Model; EHR: electronic health record; MOXXI: Medical Office of the Twenty-First Century; RAMQ: Regie de l'assurance maladie du Quebec; ID: Instructional Design; UCD: User Centered Design; IA: Information Architecture; HCI: Human Computer Interaction; AQLQ: Asthma quality of life questionnaire; TAM: Technology Acceptance Model; HRQL: health-related quality of life; IRB: institutional review board.

## Competing interests

The authors declare that they have no competing interests.

## Authors' contributions

SA conceived the study objectives, and led the design and implementation of MAP, and drafted the manuscript. SB participated in design of the MAP intervention with respect to patient-provider communication, adherence, and behaviour change. GP helped with the design of the MAP system. PE provided clinical expertise in design of the intervention. RG provided feedback on clinical integration of MAP. RP provided guidance on the design of MAP with respect to patient self-management and behaviour change. MK contributed to the design of MAP. LT participated in the design of MAP. RT participated in the design of the study including integration of MAP with existing clinical systems. All authors read and provided feedback on iterative versions of the manuscript and approved the final manuscript.
